# A Rationale for Going Back to the Future: Use of Disposable Spacers for Pressurised Metered Dose Inhalers

**DOI:** 10.1155/2015/176194

**Published:** 2015-09-27

**Authors:** Mark Sanders, Ronald Bruin

**Affiliations:** Clement Clarke International Limited, Edinburgh Way, Harlow, Essex CM20 2TT, UK

## Abstract

The introduction of pressurised metered dose inhalers (MDIs) in the mid-1950s completely transformed respiratory treatment. Despite decades of availability and healthcare support and development of teaching aids and devices to promote better use, poor pMDI user technique remains a persistent issue. The main pMDI user aid is the spacer/valved holding chamber (VHC) device. Spacer/chamber features (size, shape, configuration, construction material, and hygiene considerations) can vie with clinical effectiveness (to deliver the same dose as a correctly used pMDI), user convenience, cost, and accessibility. Unsurprisingly, improvised, low-cost alternatives (plastic drink bottles, paper cups, and paper towel rolls) have been pressed into seemingly effective service. A UK law change permitting schools to hold emergency inhalers and spacers has prompted a development project to design a low-cost, user-friendly, disposable, and recyclable spacer. This paper spacer requires neither preuse priming nor washing, and has demonstrated reproducible lung delivery of salbutamol sulphate pMDI, comparable to an industry-standard VHC, an alternative paperboard VHC, and pMDI alone. This new device appears to perform better than these other VHC devices at the low flow rates thought achievable by paediatric patients. The data suggest that this disposable spacer may have a place in the single-use emergency setting.

The introduction of pressurised metered dose inhalers (MDIs) in the mid-1950s completely transformed respiratory treatment and provoked the pace and nature of inhaler development by heralding the invention of modern dry powder and mist-type inhaler devices. Concurrent with pMDI availability, nebuliser treatment for routine respiratory care became largely a thing of the past.

The history and origins of the pMDI have been expertly and thoroughly reviewed [1, 2], and notwithstanding the ubiquity and popularity of these devices [3], commentary and research now tend to concentrate on resolution of any device-delivery shortcomings [4]. This is right and understandable: indeed, significant elements of the healthcare professional service and the respiratory treatment industry are devoted to improving the effectiveness of pMDIs in terms of both use of the best inhalation technique and drug delivered to the lung. These are interlinked issues: the plume of drug and excipients delivered (truly propelled) at high velocity from the pMDI must synchronise with the breath at the optimum point during the inhalation manoeuvre. Failure to achieve this synchronisation (hand-lung coordination) will reduce the amount of inhaled drug through throat impaction of drug particles and/or failure to carry the drug into the lung with the in-breath. With steroid-based inhaled treatments, this raises the possibility of oral side effects such as candidiasis and dysphonia [5]. These issues are much written about and familiar territory to a respiratory readership [6].

In theory—and in a perfect world—poor synchronization would be addressable via patient education, repeated practice, and clear instruction. However, despite decades of device availability and healthcare support, poor pMDI user technique remains a persistent issue. Whilst not abandoning hope, it seems that human fallibility has a significant part to play in achieving competent technique: patients and professionals alike [7–9]. In one evaluation of 150 UK healthcare professionals, only 9% could demonstrate all aspects of correct pMDI technique [10].

To address these problems, the use of a modified pMDI technique—the open mouth technique—gained some traction in the early 1980s [11, 12] and remains a taught-technique in the USA [13], with the patient actuating and inhaling whilst holding the pMDI approximately 4 cm away from the lips. Supporters point to the potential advantages of reduced oral candidiasis and improved lung deposition owing to the lower arrival-speed of the drug plume, but it is not a technique that is advocated by pMDI manufacturers: certainly not in the UK.

We have looked at this* in vitro*, comparing fluticasone propionate 250 *μ*g pMDI either with a specialized adaptor or in a conventional pMDI-impactor arrangement [14], and have also considered the effect of misalignment of the pMDI device with the open mouth, as we feel that this is an inevitable consequence of not using the sealed lips instruction. Both of these open mouth techniques were associated with reduced induction port (throat) deposition but this was attributable largely to loss to the environment. Unsurprisingly, total drug recoveries were also reduced but, in addition, the open mouth fine particle doses were markedly reduced and highly variable (Figure 1). This suggests a trade-off between potential oral side effects and drug delivery and adds confirmation that the technique particularly cannot be recommended as part of bronchodilator pMDI technique.

Concurrent with a growing realization that misuse of pMDI devices undermined their effectiveness, there was a surge in the development of teaching aids and devices to promote better instruction and/or better use [15]. These developments focused mainly on devices that, in reality, created a space between the user and the pMDI [16]—essentially a controlled version of the open mouth technique. This has been the subject of a splendid, recently published scientific history [12] that describes spacer and valved holding chamber (VHC)—essentially a valved spacer—development alongside clinical requirements and pharmacological and technological advance.

With these experiences we now have a better appreciation, particularly for valved holding chambers, of their relative merits:

- size—larger volume is better (particles airborne and available for inhalation for a longer period),
- shape—cone/pear shape is better,
- configuration—inclusion of whistles is better (flow rate determination-aid)—and inclusion of one-way valves is better (inhalation from chamber and exhalation via vents),
- material—antistatic material is better (particles remain available for inhalation),
- hygiene—antimicrobial material is better,
- convenience—small, portable, and easy to clean,
- accessibility—cost, healthcare-system funding, and patient education.

It is clear that the incentive-to-use should be, and is, a reasonable design compromise whilst maintaining the necessary effectiveness. There are now a number of commercially available holding chamber devices that include valves, collapsible volumes, whistles, antistatic and/or antimicrobial materials, and comfortable face mask interfaces (Figure 2). Recent innovations include tools to signal appropriate inspiratory flow rates, clarifying the effect of VHC whistle-position [17], combining antistatic/antimicrobial materials [18], and awareness of drug wastage on VHC face mask materials [19].

VHCs, in particular, are a preeminent advance (i) for improving pMDI drug delivery for young and very young children, especially for those whose preference or ability is for receiving drug via tidal breaths, (ii) for treatment, emergency department admission, and recovery-time for acute asthma, and (iii) for inhaled steroid use in children. Internationally, this is now reflected in asthma guidelines which recommend their use [20–22]. With regard to performance comparison, it is important to remember that the role of a spacer or VHC is to deliver the same dose as a correctly used pMDI, that is, the dose that has been validated through clinical study for safety and efficacy. It would be easy to design a device that improved pMDI performance but in doing so the known safety and efficacy boundaries would have been abandoned. VHC/spacer design is, therefore, about matching performance. Performance is measured* in vitro*, using laboratory cascade impactors and vacuum pumps that reflect adult inhalation forces, typically 28–30 L/min, with a performance of ±15% of the correctly used pMDI as the basis for determining equivalence. It is interesting that these approval requirements do not consider the inhalation flow rates of the paediatric population, probably the main user-group.

It goes without saying but bears repeating that the thoroughly considered treatment and management strategies espoused in guidelines should be followed wherever possible. However, local considerations—access to and availability of treatments, economics, medical facilities, healthcare support, and so forth—will affect use and adherence. This has led to inventive alternatives and to the consideration of influential work which provided some of the bases for the development of VHCs. Unsurprisingly the use of improvised, low cost spacer devices virtually paralleled the development of pMDIs [12]. Over the years, plastic drink bottles, paper cups, Styrofoam cups, and paper towel rolls have all been pressed into service.

What would a user want from a self-sourced/home-made device? Certainly low cost, ease of use, and ready availability, plus as many of the desirable features of a commercial VHC as possible: free from contaminants, collapsible (disposable?), a good interface between the device and the mouth [23], and low susceptibility to static, transparent, and, of course, effective. A recent study has attempted to determine the very important aspect of effectiveness of these “nonconventional” devices when paired with beclometasone pMDI and compared with the AeroChamber VHC and with the open mouth pMDI technique [24]. All bar one of the nonconventional devices—the nebuliser reservoir tubing—performed very well (throat deposition, fine particle fraction) and at least as well as the AeroChamber. This is an important piece of research but it must be borne in mind that these were* in vitro* tests. Nevertheless,* in vitro* data remain the cornerstone of respiratory and comparative inhalation research and cannot therefore be dismissed because the test components of the research were unconventional.

In the UK, the law is changing with regard to the scholastic approach to children with asthma [25]. From October 1, 2014, all primary and secondary schools have been allowed to hold spare emergency inhalers, if they choose to do so. Guidance has been [26] or is in the process of being issued. In England [27], an asthma emergency kit should include a salbutamol pMDI and at least two single-use plastic spacers compatible with the inhaler, plus all appropriate instructions and checklists. The guidance is nonstatutory but it is clear that the expectation is use of the pMDI with a spacer (i.e., VHC), but with the usual domestic requirement to clean and air-dry the VHC replaced by disposability to ensure good hygiene.

Encouraged by this, we have looked again at the features of self-sourced or readily disposable VHCs. To the requirements listed above, we have added familiarity and performance reliability. The majority of the use of these devices will be in an emergency situation, in unfamiliar surroundings, possibly away from home, or remote from facilities suitable to maintain device hygiene. The device should therefore be as close to self-explanatory to use as possible and create minimal waste on disposal.

There is an extensive literature on home-made spacers, plus a Cochrane review comparing the acute therapy response in children to inhaled *β*_2_ agonists delivered via pMDI using home-made spacers with the use of commercially produced spacers, relating to acute exacerbations of wheezing or asthma [28]. The Cochrane requirements (study, participant, and intervention) were rigorous, resulting in only six included studies. The review did not identify a difference between home-made and commercial devices but emphasized that, owing to database size, neither could equivalence be claimed. Interestingly, five of the studies used plastic drink bottles, with one study also including a polystyrene cup [29] (which was the least effective), and the sixth, an unpublished study, utilizes a cardboard cone. Other randomized comparative clinical trials have led to more positive views: with no differences with a 150 mL paper disposable cup [30] or with plastic bottles [29–33]. The consensus seems clear, however, that use of a commercially made spacer device is, at all times, preferable to the use of a home-made device* except* where emergency situations or hygiene considerations dictate otherwise (and a commercially produced spacer is not available), with reduced spacer volume and absence of a valve being acceptable sacrifices in terms of dose delivery and ease of tidal breathing [30, 34, 35].

Revisiting the teachings of the literature and accepting that a single-use valved spacer would be neither cost-sensitive to consumers nor readily disposable, we have devised a paper-based, fully disposable spacer that includes both a secure fit for the pMDI and a mouthpiece feature. Despite the preferred cone shape of a spacer, the pMDI actuator outlet (pMDI mouthpiece-spacer inlet) and the outlet to the patient (mouthpiece) are identical in design and construction (Figure 3). This has advantages in terms of production and cost, packaging, storage and transportation, and familiarity and ease of assembly for the end-user (patient and/or carer). The use of a paper “space” has the advantage of being nonelectrostatic. The most efficient method of neutralizing charge on device plastics is washing in a soapy water solution followed by air drying, with the soapy water acting as a surfactant. An alternative is to deliberately allow the charged medication particles to “soak-up” the electrostatic charge: the GINA guidelines [21] refer to needing to use at least 20 puffs into a new unwashed chamber before delivery of a rescue dose. Neither method is practical (or necessary) for a paper-based disposable device and/or for emergency use straight from the packaging.

Preliminary* in vitro* research with the DispozABLE Spacer has been carried out comparing salbutamol sulphate delivery from Ventolin HFA pMDI (GSK, 90 *μ*g ex-mouthpiece, 108 *μ*g ex-valve) alone (*n* = 5) and with the new spacer (*n* = 3) [36]. Devices were attached to a Next Generation Impactor, with drug, impactor, and devices used according to manufacturers' instructions and regulatory methodology. In addition to standard actuation procedure, data were also collected when a one-second delay was introduced between actuation and impactor function to mimic suboptimal use conditions. Particle size and dose fractions (% valve label) were determined (Table 1). The mean fine particle doses from the pMDI alone and from pMDI plus DispozABLE Spacer were very similar and comparable to conventional valved spacers. When used suboptimally (easily envisaged in nonroutine and emergency situations), the pMDI plus DispozABLE Spacer performed much better than the pMDI alone, delivering 37.7 *μ*g.

Additional* in vitro* impactor data have demonstrated inter- and intrasample comparability [36] and no significant differences in respirable and fine particle salbutamol sulphate doses between the DispozABLE Spacer, an industry-standard VHC device OptiChamber Diamond (Philips Respironics), and the Ventolin pMDI alone [37].

We are aware of one other paperboard disposable spacer, the Thayer Medical LiteAire collapsible VHC spacer [38], which is more complex in construction and has a chamber capacity of 160 mL (DispozABLE Spacer, 230 mL). A controlled clinical trial of the LiteAire plus pMDI in emergency room bronchodilator treatment of acute asthma [39] found it not inferior to nebulised therapy and considerably less costly. We have now compared salbutamol sulphate delivery, at a 12 L/min flow rate representative of paediatric use, via DispozABLE Spacer and OptiChamber Diamond [39] and, in a separate experiment, via the LiteAire. In both experiments, the DispozABLE Spacer was significantly better than both comparative VHCs and delivered a performance similar to pMDI alone.

A review of the posts and responses on the mumsnet by parents for parents website reveals the extent of confusion and misunderstanding around the correct use of inhaler devices, despite medicoclinical advice and literature [40]. We see a place for a clean (free from contaminants), low-cost, environmentally friendly, disposable spacer in those clinical situations where effective use directly from the packaging is desirable: pulmonary function reversibility testing, ambulance use, emergency and isolation room use, and in the lay environment as part of the new school emergency inhaler toolkit and as a household emergency backup device.

## Figures and Tables

**Figure 1:**
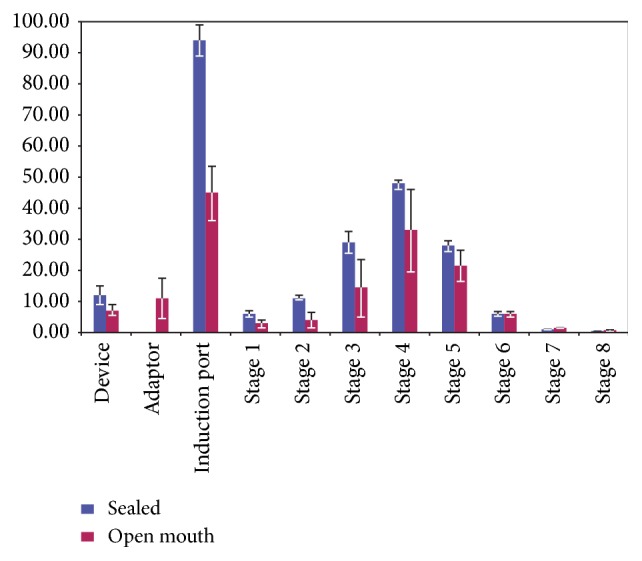
Aerodynamic size distribution (mean ± SD) of a 250 *μ*g fluticasone propionate pMDI (Flixotide Evohaler) at 30 L/min: open (*n* = 6); sealed (*n* = 4).

**Figure 2:**
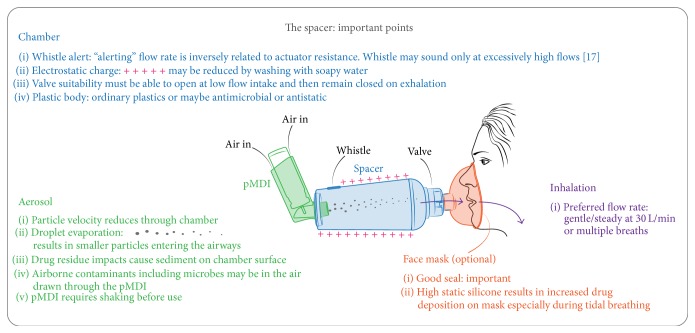
Spacer features, drug formulation, and in-use technique can affect pMDI drug delivery.

**Figure 3:**
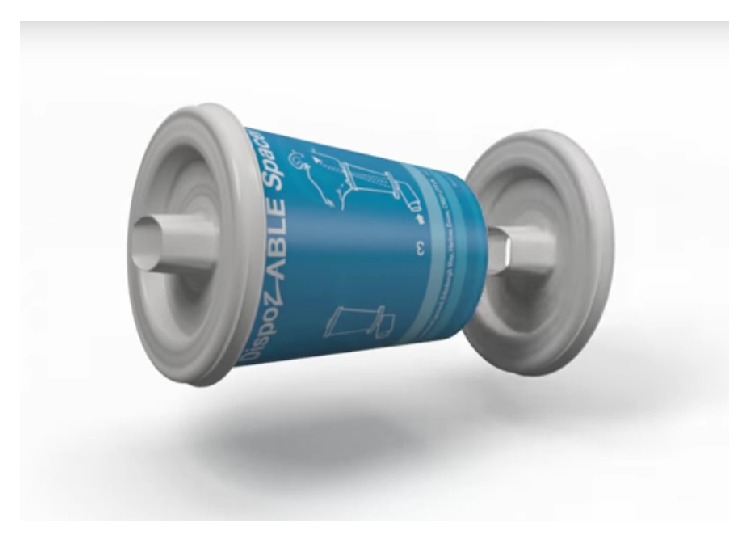
DispozABLE Spacer.

**Table 1:** Fine particle dose data (particle size <5 *μ*m, mean *μ*g ± standard deviation).

pMDI + spacer device	Fine particle dose	
	(mean *μ*g ± standard deviation)	
	Optimal use	Suboptimal use
pMDI alone (*n* = 5)	55.1 ± 4.55	10.2 ± 2.17
pMDI + DispozABLE Spacer	56.6 ± 5.65	37.7 ± 7.65
